# Cratoxylumxanthone C, a natural xanthone, inhibits lung cancer proliferation and metastasis by regulating STAT3 and FAK signal pathways

**DOI:** 10.3389/fphar.2022.920422

**Published:** 2022-08-09

**Authors:** Yeling Li, Huimei Wang, Wenhui Liu, Jiantong Hou, Jing Xu, Yuanqiang Guo, Ping Hu

**Affiliations:** ^1^ State Key Laboratory of Medicinal Chemistry Biology, College of Pharmacy and Tianjin Key Laboratory of Molecular Drug Research, Nankai University, Tianjin, China; ^2^ Key Laboratory of Tropical Medicinal Resource Chemistry of Ministry of Education, Hainan Normal University, Haikou, China; ^3^ Key Laboratory of Research on Pathogenesis of Allergen Provoked Allergic Disease in Liaoning Province, Shenyang Medical College, Shenyang, China

**Keywords:** cratoxylumxanthone C, anti-tumor, stat3, FAK, natural xanthone, zebrafish

## Abstract

To discover phytochemicals as lead compounds for cancer treatment, cratoxylumxanthone C, a natural xanthone, was obtained from *Cratoxylum cochinchinense* (Lour.) Bl., for which there have been no reports on the biological effects against cancer*.* Our study revealed that cratoxylumxanthone C had significant anti-tumor activity by inducing apoptosis, augmenting cellular reactive oxygen species (ROS), and arresting cell circle. The mechanistic examination showed the inhibition of A549 cell proliferation and metastasis by cratoxylumxanthone C was coupled with the signal transducer and activator of transcription 3 (STAT3) and focal adhesion kinase (FAK) signaling pathways. Furthermore, the zebrafish models confirmed its significant *in vivo* anti-tumor activity, in which cratoxylumxanthone C inhibited tumor proliferation and metastasis and suppressed the angiogenesis. Comprehensively, these cellular and zebrafish experiments implied that cratoxylumxanthone C may have the potential to become an anti-tumor agent for lung cancer, especially non-small cell lung cancer (NSCLC).

## Introduction

Lung cancer, accounting for about 11.4% of all cancers, is a common malignant tumor all over the world (Sung et al., 2021). Lung cancer can be divided into two categories, small cell lung cancer (SCLC) and non-small cell lung cancer (NSCLC) according to different growth and diffusion modes (Lemjabbar-Alaoui et al., 2015). NSCLC accounts for over 80% of lung cancers and is the most common and deadly form of primary lung cancer (Singh et al., 2021). Currently, surgery, radiotherapy, and chemotherapy have been considered as common treatments for NSCLC. Despite great progress in the diagnosis and therapy of NSCLC, the 5-year survival rate of NSCLC is less than 20% (Cui et al., 2022). Therefore, there is an urgent need to develop new and more effective strategies for the treatment of NSCLC.

**FIGURE 1:**
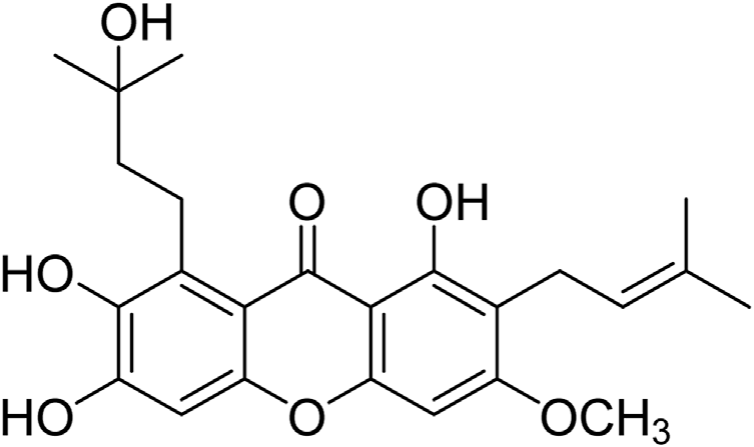
Chemical structure of cratoxylumxanthone C.

Natural drugs mainly from plants are important weapons for human beings to fight against diseases, in which many natural bioactive products have been found as potential drug molecules. Natural products play a key role in cancer chemotherapy and chemoprevention. Some famous drugs including paclitaxel, camptothecin, and vincristine are derived from phytochemicals or their derivatives (Huang et al., 2018). Plants or natural products from plants have made important contributions to the treatment of human cancers. As a deciduous shrub or tree, *Cratoxylum cochinchinense* belongs to the Clusiaceae plant family, in which some species have been used historically as traditional medicines (Duan et al., 2012). Previous phytochemical investigations have indicated that the plants of *Cratoxylum* genus are rich in xanthones with extensive biological activities including anti-malaria, anti-bacteria, anti-HIV, and cytotoxicity (Laphookhieo et al., 2006; Laphookhieo et al., 2009; Rattanaburi et al., 2014; Duan et al., 2015; Ito et al., 2017; Li et al., 2018; Huang et al., 2019; Jia et al., 2019; Lv et al., 2019). Some xanthones, e.g., gambogic acid, were found to have promising cytotoxic effects and be potentially useful for the discovery of anticancer lead compounds. The unique structures and notable cytotoxic activity of xanthones have attracted our great interest. To obtain bioactive xanthones, our research group investigated the chemical constituents of some *Cratoxylum* species rich in xnathones. In our phytochemical investigation, cratoxylumxanthone C (Figure 1) was purified from *C. cochinchinense*, and the biological screening showed that cratoxylumxanthone C had significant cytotoxic effects toward to some human cancer cell lines, especially A549 cell line. According to the literature on this natural product reported previously, there have been no reports on the biological effects against cancer and the underlying mechanistic study of cratoxylumxanthone C, which prompted us to investigate the potential of this natural product as lead compound to combat cancer.

Considering the severity of lung cancer, in particularly NSCLC, and the strong cytotoxic effects of cratoxylumxanthone C on A549 cells, this study aims to reveal its anti-tumor mechanism and explore its development potential based on the *in vivo* antitumor experiments. The signaling pathways of transcription 3 (STAT3) and focal adhesion kinase (FAK) affected by cratoxylumxanthone C, which were closely coupled with tumor proliferation and migration, were investigated. While, the angiogenesis, a pivotal sign of tumor progression, was also explored by utilizing transgenic zebrafish models.

## Materials and methods

### Materials and cell culture

Materials are appended in the Supplementary Material. Cell culture (A549, HepG2, and MCF7 cells) was performed as stated in the Supplementary Material.

### MTT assay

*In vitro* anti-tumor activity of cratoxylumxanthone C was detected by MTT assay (Zhou et al., 2021). Briefly, A549, HepG2 and MCF7 cells were inoculated in 96-well plates at a density of 5 × 10^3^ cells/well, and cultured under appropriate conditions (5% CO_2_, 37°C) for 24 h. The cells were treated with cratoxylumxanthone C for 48 h, and then 20 *μ*L of 3-(4,5-dimethylthiazol-2-yl)-2,5-diphenyltetrazolium bromide (MTT) solution (5 mg/mL) was added to the wells and incubated at 37°C for 4 h. After removing the solution, 150 *μ*L of dimethyl sulfoxide (DMSO) solution was added and the optical density value was recorded at 492 nm using a microplate reader (Thermo Fisher Scientific, Waltham, MA, United States). Each experiment was repeated three times. The inhibition rates were analyzed by CompuSyn software (CompuSyn, NJ) and presented as IC_50_ value.

### Cell apoptosis detection

The detection of cell apoptosis affected by cratoxylumxanthone C was accomplished by flow cytometry (Li et al., 2021a; Zhang et al., 2021a). Briefly, A549 cells were harvested and seeded in 12-well plates (1 × 10^5^ cells per well) and incubated for 24 h at 37°C. Then, cells were treated with cratoxylumxanthone C (7.5, 15, and 30 *μ*M) for 48 h. After incubation for 48 h, the cells were harvested, washed twice with PBS, and resuspended with binding buffer (Beyotime, Shanghai, China). Subsequently, Annexin V-FITC (5 *μ*L) and PI (10 *μ*L) were added, and the suspension was incubated in the dark at room temperature for 20 min. Then, cell apoptosis was detected by BD LSRFortessa flow cytometer (BD Biosciences). The data were processed with FlowJo flow cytometry analysis software.

### Meaurement of ROS release

The intracellular ROS realease was measured by flow cytometer (Ye et al., 2021; Ma et al., 2022). Briefly, A549 cells were inoculated in 12-well plates (1 × 10^5^ cells per well) and cultured under appropriate conditions (5% CO_2_, 37°C) for 24 h. Then, A549 cells were treated with cratoxylumxanthone C (7.5, 15, and 30 *μ*M) for 48 h. Then, the cells were collected, washed with PBS, and stained with 10 *μ*M DCFH-DA probe (Beyotime, Shanghai, China) for 20 min at 37°C. Finally, the cells were washed three times with serum-free culture medium and subjected to the flow cytometer (BD Biosciences). The ROS release was analyzed according to the FlowJo flow cytometry analysis.

### Cell cycle analysis

The effects of cratoxylumxanthone C on A549 cell circle were evaluated by flow cytometric analysis (Han et al., 2019; Zhao et al., 2022). In brief, A549 cells were inoculated in 12-well plates (1 × 10^5^ per well), and cultured under normal conditions (5% CO_2_, 37°C) for 24 h. Then, the cells were treated with cratoxylumxanthone C (7.5, 15, and 30 *μ*M) and incubated for 48 h. At the end of incubation, the cells were harvested, washed with PBS, and fixed with 70% ice cold ethanol at 4°C overnight. Subsequently, the cells were washed with PBS and stained with RNase-containing propidium iodide staining buffer for 30 min at 37°C in the dark. Cellular DNA analysis was conducted by BDLSR Fortessa flow cytometer (BD Biosciences). Data were processed by ModFit LT Software.

### Wound-scratch assay

The effects of cratoxylumxanthone C on A549 cell migration were evaluated by wound scratch assay (Sinagra et al., 2015). Briefly, A549 cells were inoculated in 6-well plates (5 × 10^5^ per well) and cultured under appropriate conditions (5% CO_2_, 37°C) for 24 h. Then, cells were scratched with a sterile pipette tip, and incubated with cratoxylumxanthone C (7.5, 15, and 30 *μ*M) for 48 h. The wound healing at 0 and 48 h after the scratch was observed with a microscope, and the migration rate was measured with ImageJ software (NIH, Bethesda, Maryland, United States).

### Western blotting experiments

A549 cells were seeded in 6-well plates (2 × 10^5^ per well) and cultured under normal conditions for 24 h. Cratoxylumxanthone C was added and the cells were incubated continuously for 48 h. At the end of incubation, the cells were washed with cold PBS, and lysed with Radio Immunoprecipitation Assay (RIPA) lysis buffer containing 1 mM phenylmethylsulfonyl fluoride (PMSF). The cells were collected, shaken for 30 min at 4°C, and centrifuged at 12,000 rpm for 10 min at 4°C, and the total proteins were extracted. Protein concentrations were measured with BCA Protein Assay Kit (Beyotime, P0012S). The proteins were subjected to 12% SDS-PAGE electrophoresis and then transferred to polyvinylidene difluoride (PVDF) membrane. The membrane was blocked with TBST (TBS containing 1%-tween 20) solution containing 5% skim milk for 60 min at room temperature, and incubated with primary antibodies (*β*-actin, STAT3, p-STAT3, cyclin D1, caspase-3, caspase-9, Bcl-2, FAK, p-FAK, Mcl-1 and MMP-2) at 4°C overnight. The membranes were washed with TBST, and then incubated with secondary antibody for 1 h. The protein blots were developed with ECL Detection Kit. Each strip was quantified by ImageJ software.

### Zebrafish breeding

Details for zebrafish feeding and breeding are described in the Supplementary Material.

### *In vivo* anti-tumor assay using zebrafish xenograft model

Zebrafish xenograft tumor model was used to study the inhibition of cratoxylumxanthone C on tumor proliferation and invasion. Zebrafish xenografts were established according to previously reported methods (Li et al., 2016; Zhang et al., 2021b). In brief, A549 cells were stained with 2 *μ*M CM-DiI and suspended in serum-free culture medium (1 × 10^7^ cells/mL). Then, 48 h post fertilization (hpf) embryos were anesthetized with 0.02% tricaine solution, and 5 nL of labeled A549 cell suspension was microinjected into the yolk sac of embryos. The embryos were incubated continuously at 28.5°C for 4 h and subsequently treated with cratoxylumxanthone C (2.5, 5, and 10 *μ*M). After incubation with the compound for 48 h, the proliferation and metastasis of A549 cells were observed with laser confocal microscope (Leica, TCS SP8, Germany) and quantitatively processed with ImageJ software.

### *In vivo* antiangiogenetic assay

The angiogenesis inhibition of cratoxylumxanthone C was assessed with the transgenic zebrafish *Tg(fli1: EGFP)* model (Zhang J. et al., 2021; Huang et al., 2015). Briefly, 6 hpf zebrafish embryos were administrated with cratoxylumxanthone C (5, 10, and 20 *μ*M) and incubated continuously for 48 h. At the end of incubation, embryos were anesthetized with 0.02% tricaine and the development of intersegmental vessels (ISVs) was detected by confocal microscope (Leica, TCS SP8, Germany). The length of ISVs was quantified by ImageJ software.

### Statistical analysis

Data were analyzed by GraphPad Prism 6.0 (GraphPad, Software, San Diego, CA, United States). Statistical comparison was performed by one-way ANOVA. Probabilities (*p*) less than 0.05 were determined to be significant.

## Results

### Cratoxylumxanthone C inhibited cancer cells growth *in vitro*

**FIGURE 2:**
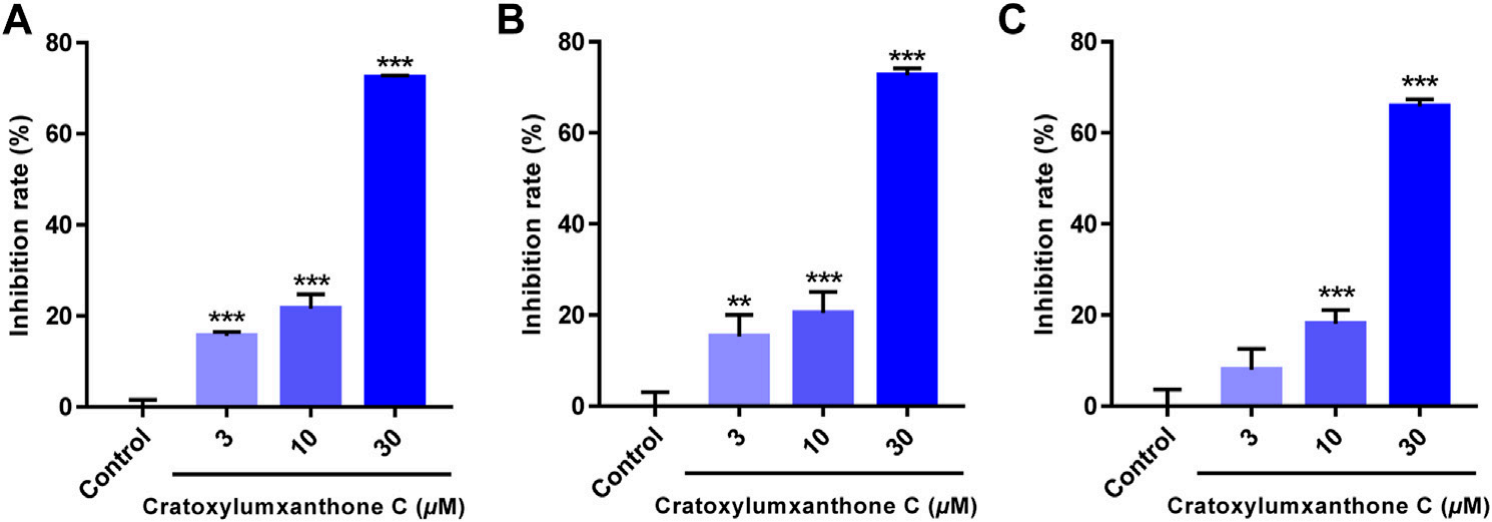
Cratoxylumxanthone C inhibited the proliferation and viability of three human cancer cell lines. A549 **(A)**, HepG2 **(B)** and MCF7 **(C)** cells were treated with cratoxylumxanthone C (3, 10, and 30 *μ*M) for 48 h. Cell viability was detected by MTT assay. The results are presented as means ± SD. ^**^ *p* < 0.01 and ^***^ *p* < 0.001 versus control group.

Cratoxylumxanthone C was obtained from the *C. cochinchinense*. The experimental results of the MTT assay illustrated that cratoxylumxanthone C exhibited significant antiproliferative effects on A549, HepG2, and MCF7 cells, which inhibited tumor cells proliferation dose-dependently (Figures 2A–C). The IC_50_ values were collated in Table 1, which showed cratoxylumxanthone C had strong inhibitory effects when compared with etoposide. As shown in Table 1, the IC_50_ values of the three cell lines were calculated, and the results showed in contrast with the positive control etoposide, cratoxylumxanthone C seemed to have more inhibitory effects on A549.

**TABLE 1:** Cytotoxicities of cratoxylumxanthone C against three human cancer cell lines.

Compound	IC_50_ (*μ*M)		
	A549	HepG2	MCF7
Cratoxylumxanthone C	17.5 ± 1.0	17.7 ± 1.9	21.9 ± 0.7
Etoposide^a^	33.8 ± 1.0	1.4 ± 0.5	28.6 ± 1.1

a Etoposide was used as a positive control. All results are presented as means ± SD.

### Cratoxylumxanthone C induced apoptosis of A549 cells

**FIGURE 3:**
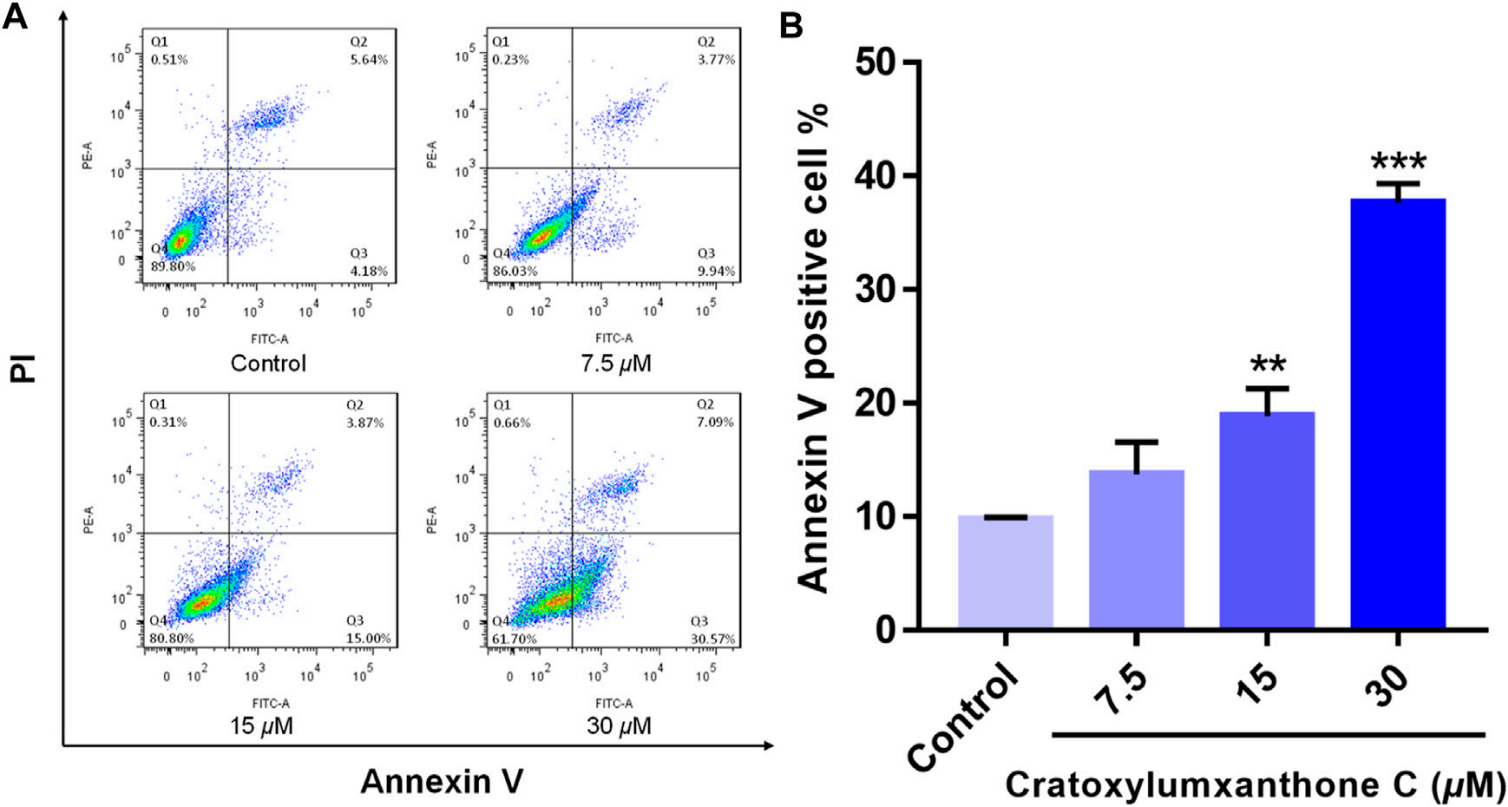
Cratoxylumxanthone C induced apoptosis in A549 cells. A549 cells were treated with different concentrations (7.5, 15, and 30 *μ*M) of cratoxylumxanthone C for 48 h. Cells were stained with Annexin V and propidium iodide (PI), and subsequently analyzed by flow cytometry. **(A)** Flow cytometric analysis of A549 cells after treated with different concentrations of cratoxylumxanthone C. **(B)** Histogram of the proportions of apoptotic cells at 48 h with the treatment of cratoxylumxanthone C. The results are expressed as means ± SD. ^**^ *p* < 0.01 and ^***^ *p* < 0.001 versus control group.

To examine whether cratoxylumxanthone C suppressed the proliferation of A549 cells *via* inducing apoptosis, the apoptosis detection induced by cratoxylumxanthone C was performed. A549 cells were administrated with different doses of cratoxylumxanthone C (7.5, 15, and 30 *μ*M) and then apoptosis was detected with Annexin V-FITC and PI staining. As shown in Figure 3, with the concentration increase of cratoxylumxanthone C, the percentage of apoptotic cells rose from 9.82% (control) to 13.71% (7.5 *μ*M), 18.87% (15 *μ*M), and 37.66% (30 *μ*M). These results showed that cratoxylumxanthone C could inhibit the cell proliferation by inducing cell apoptosis in A549 cells.

### Cratoxylumxanthone C increased cellular ROS

**FIGURE 4:**
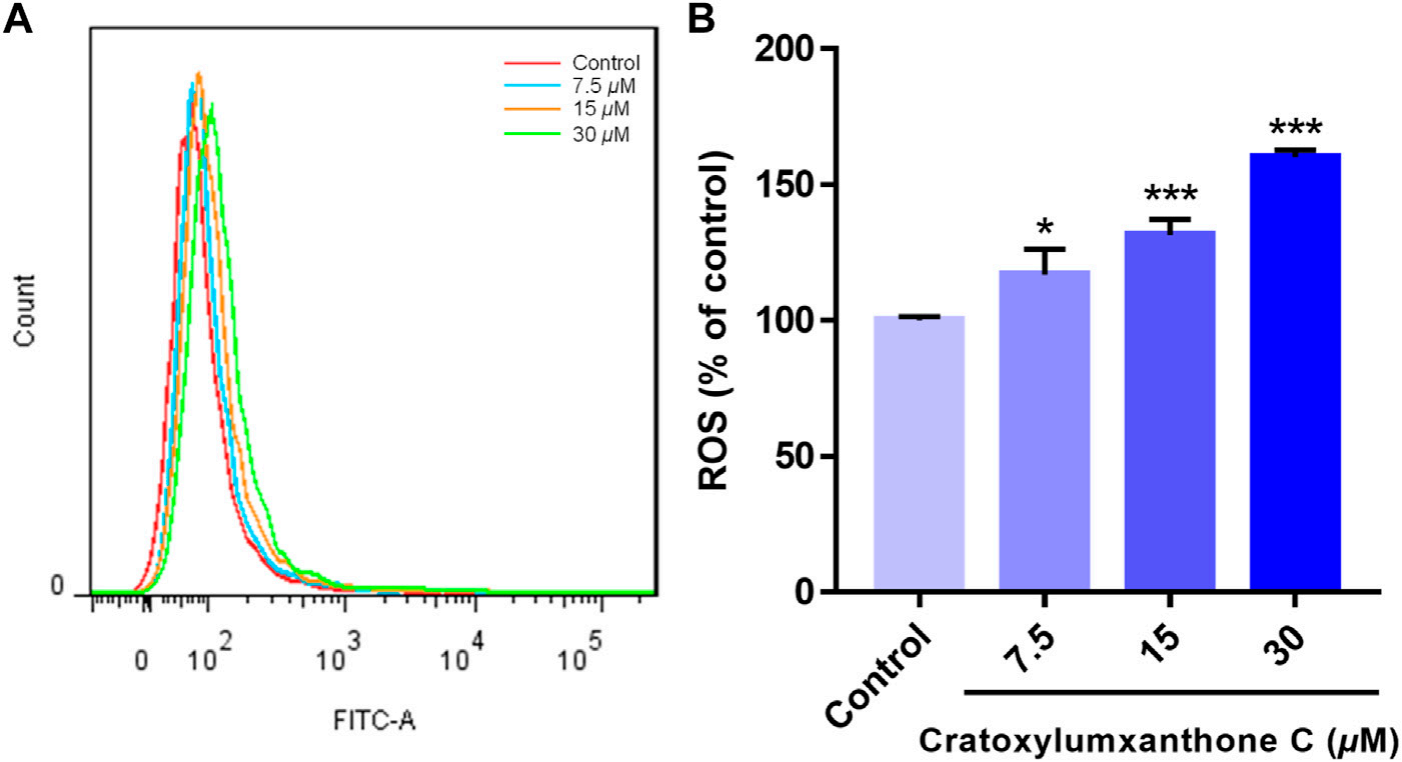
Cratoxylumxanthone C increased ROS production in A549 cells. A549 cells were treated with different concentrations (7.5, 15, and 30 *μ*M) of cratoxylumxanthone C for 48 h. Cells were stained with DCFH-DA, and analyzed by flow cytometer. **(A)** Flow cytometric analysis of A549 cells after treated with different concentrations of cratoxylumxanthone C. **(B)** Histogram of relative ROS level compared with control group. The results are expressed as means ± SD. ^*^ *p* < 0.05 and ^***^ *p* < 0.001 versus control group.

ROS level has been reported to be a crucial indicator in cell apoptosis, and the increase of ROS can promote apoptosis. To further observe and confirm the effects of cratoxylumxanthone C on the levels of ROS, A549 cells were administrated with cratoxylumxanthone C (7.5, 15, and 30 *μ*M), and then stained with DCFH-DA probe. As illustrated in Figure 4, cratoxylumxanthone C significantly increased ROS level in A549 cells. As the concentrations of cratoxylumxanthone C rose, the ROS levels of A549 cells were 1.17 (7.5 *μ*M), 1.31 (15 *μ*M) and 1.60 (30 *μ*M) times higher than that of the control group. The ROS detection revealed that cratoxylumxanthone C could increase the production of ROS.

### Cratoxylumxanthone C arrested A549 cell cycle

**FIGURE 5:**
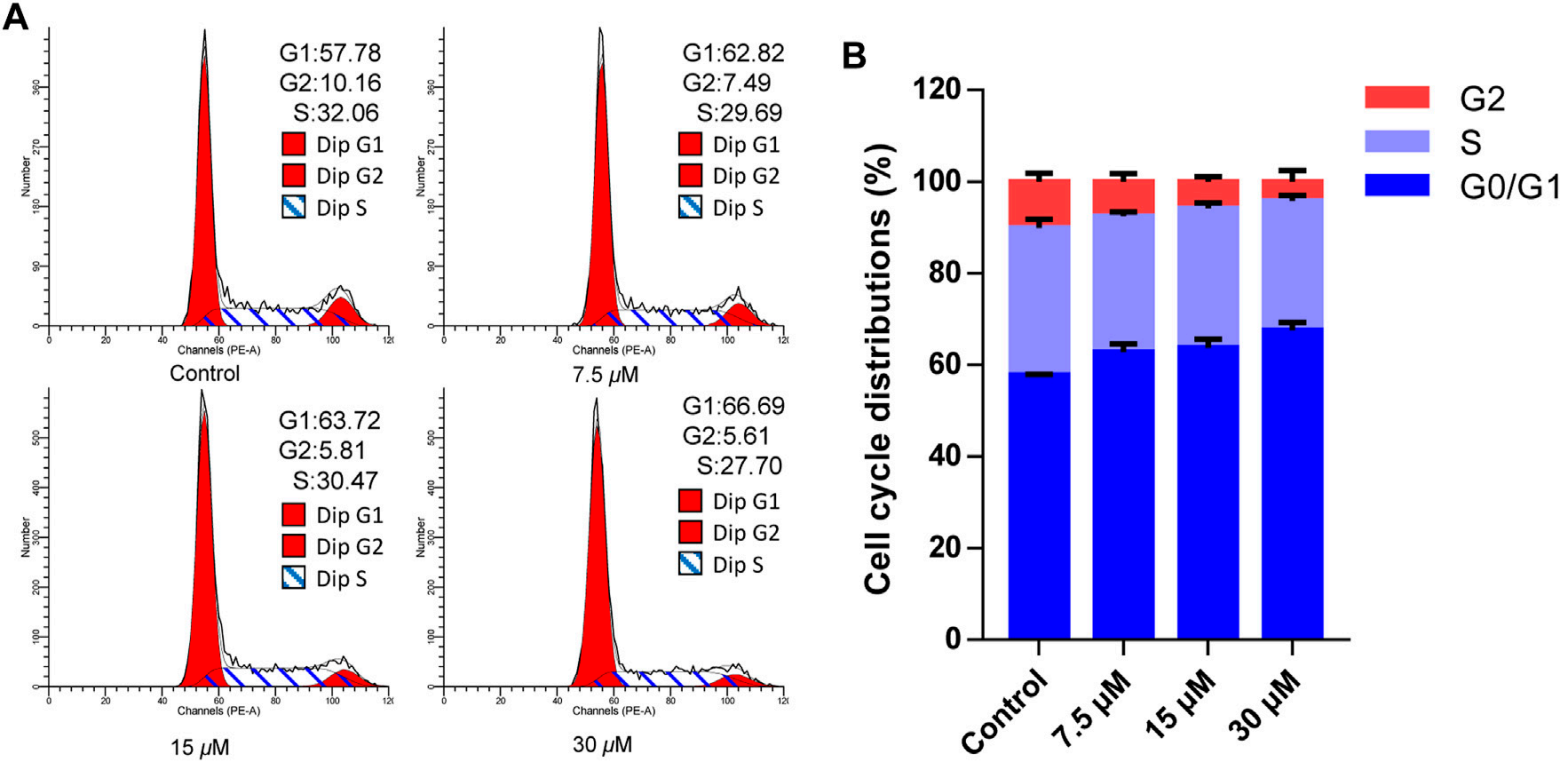
Cratoxylumxanthone C arrested G0/G1 phase in A549 cells. A549 cells were treated with different concentrations (7.5, 15, and 30 *μ*M) of cratoxylumxanthone C for 48 h. Then the cells were stained with propidium iodide (PI), and the cell cycle distribution was analyzed using flow cytometry. **(A)** Flow cytometric analysis of A549 cells after treated with different concentrations of cratoxylumxanthone C. **(B)** Histogram of cell cycle phases distribution.

Cell apoptosis was intimately related to cycle arrest, so the cell cycle effects affected by cratoxylumxanthone C were examined. After administrated and stained with PI, the cell cycle arrest was analyzed by flow cytometry. As preseneted in Figure 5, A549 cells treated with cratoxylumxanthone C were arrested in G0/G1 phase. When the concentration increased from 0 to 30 *μ*M, the proportion in G1 phase rose from 57.78% (control) to 62.82% (7.5 *μ*M), 63.72% (15 *μ*M), and 66.69% (30 *μ*M), while the percentage in S and G2 phase decreased. The data indicated that cratoxylumxanthone C could arrest cell circle in G0/G1 phase.

### Cratoxylumxanthone C affected the expression of apoptosis-related proteins

**FIGURE 6:**
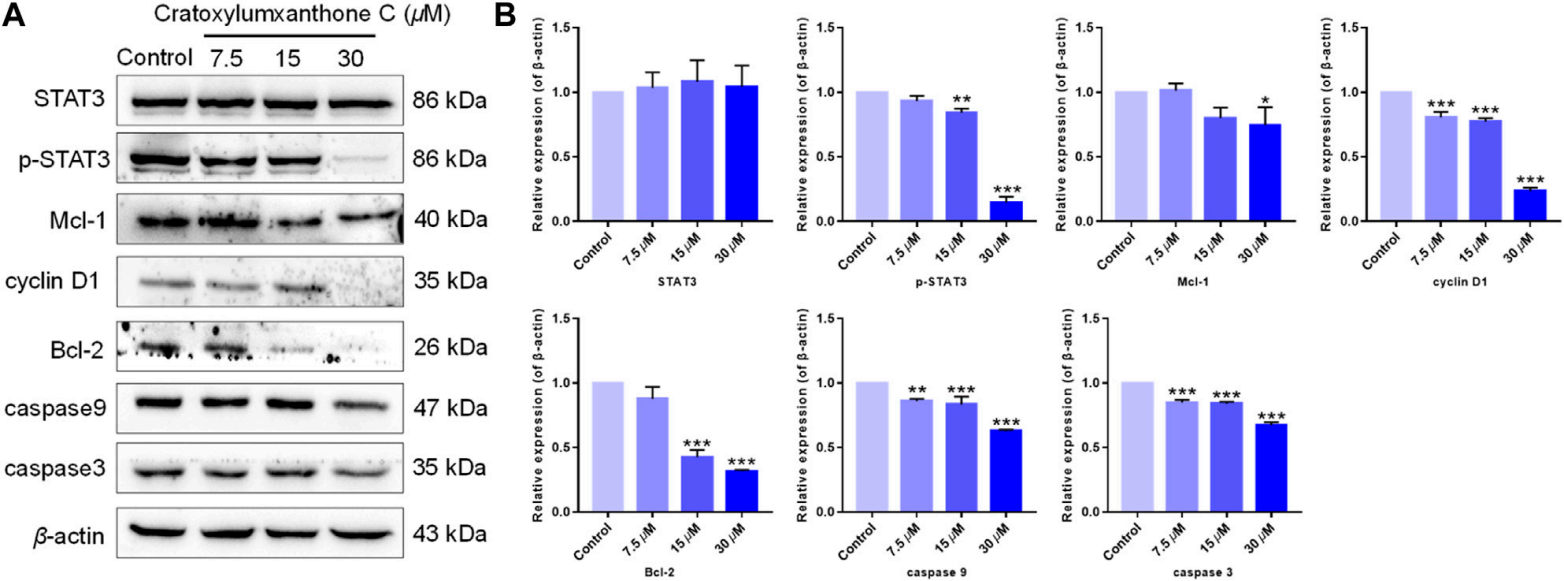
Cratoxylumxanthone C regulated STAT3 signaling pathway and apoptosis-related proteins. **(A)** Western blotting analysis was conducted to evaluate the effects of cratoxylumxanthone C on the expression of STAT3, p-STAT3, Mcl-1, cyclin D1, Bcl-2, caspase-3, and caspase-9. **(B)** Histogram of the protein relative expression level compared with control group. The results are expressed as means ± SD. *p < 0.05, **p < 0.01 and ***p < 0.001 versus control group.

**FIGURE 7:**
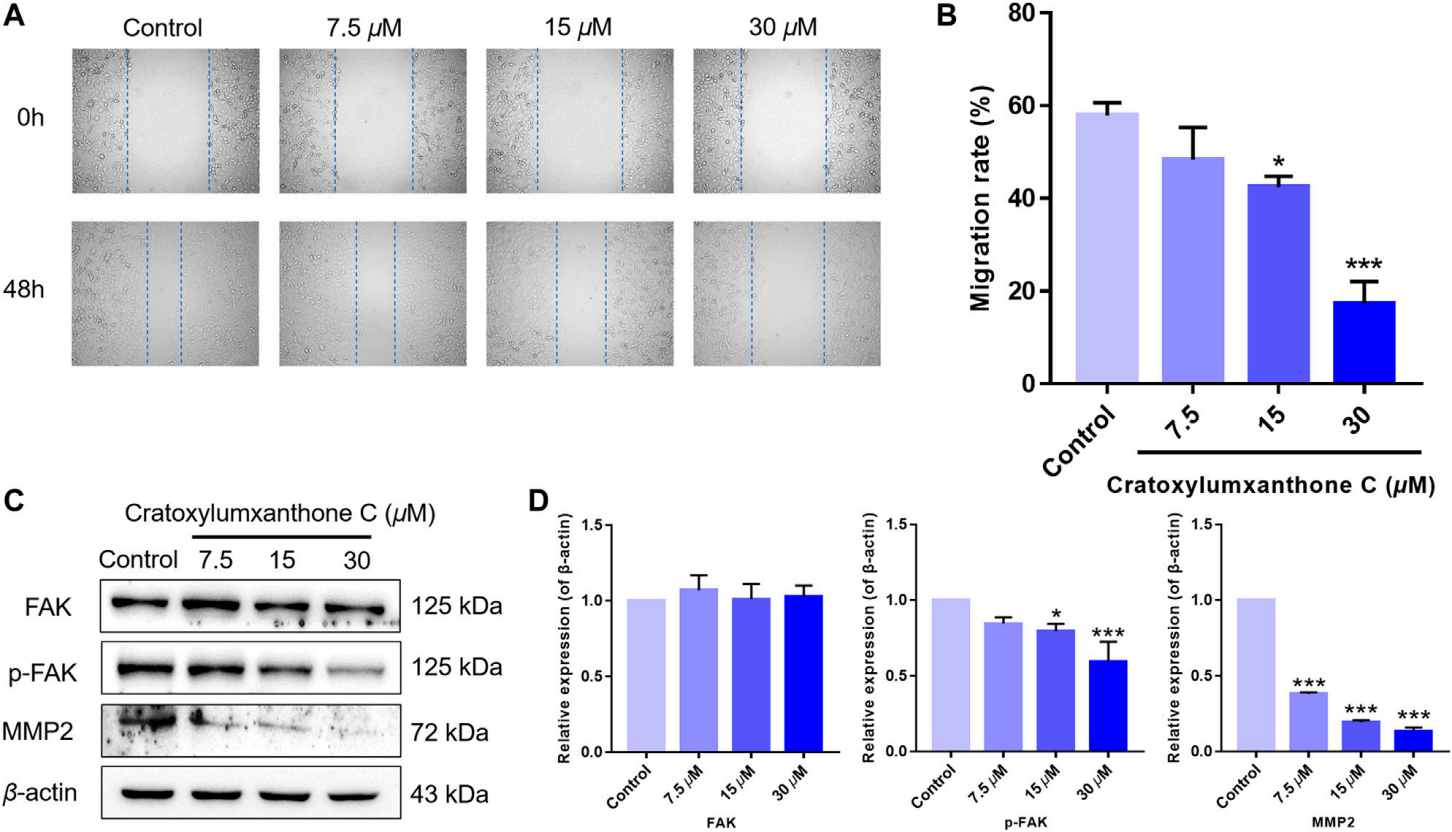
Cratoxylumxanthone C inhibited migration in A549 cells and regulated FAK/MMP2 signaling pathway. **(A)** A549 cells were photographed at 0 and 48 h. **(B)** Data of migration rates (%) were shown in the histogram. **(C)** Western blotting analysis was used to evaluate the effects of cratoxylumxanthone C on the expression of FAK, p-FAK, MMP2. **(D)** Histogram of the protein relative expression level compared with control group. The results are expressed as means ± SD. *p < 0.05 and ***p < 0.001 versus the control group.

The cell cycle distribution and ROS detection indicated that cratoxylumxanthone C blocked the cell cycle and induced the accumulation of ROS dose-dependently. Normally, ROS increase and cell cycle arrest influenced the levels of apoptosis-related proteins, leading to apoptosis. As shown in Figure 6, after treatment with cratoxylumxanthone C, the expression levels of Bcl-2 protein in A549 cells were obviously decreased with the concentration increase of cratoxylumxanthone C. In addition, the downstream proteins of cell cycle and ROS-dependent pathways, cyclin D1, caspase-9, and caspase-3 affected by this compound were also examined. As shown in Figure 7, we further confirmed that the cyclin D1, caspase-9, and caspase-3 was decreased in A549 cells after treatment with cratoxylumxanthone C. These results revealed that cratoxylumxanthone C induced apoptosis by stimulating ROS and blocking cell cycle.

### Cratoxylumxanthone C regulated STAT3 signaling pathway

Tumor proliferation is regulated by numerous genes or proteins, of which STAT3 and the downstream proteins participate in regulation. In order to further confirm the mechanism of by which cratoxylumxanthone C inhibited cell proliferation, the expression of a few proliferation-related proteins after treatment with cratoxylumxanthone C was detected. According to the Western blotting analysis, cratoxylumxanthone C did not affect the expression of STAT3 (Figure 6), but the phosphorylation of STAT3 at Tyr 705 was decreased dose-dependently (Figure 6). Then, the levels of cyclin D1, Mcl-1 and Bcl-2 downstream of STAT3 were detected, which showed that the cell cycle regulator cyclin D1, the anti-apoptotic protein Bcl-2 and Mcl-1 decreased dose-dependently manner from the Western blotting experiments (Figure 6). These findings demonstrated that cratoxylumxanthone C exerted anti-prolieration activity in A549 cells by regulating STAT3 pathway.

### Cratoxylumxanthone C regulated FAK/MMPs signaling pathway

Migration is another important feature of tumors. Thus, we performed wound healing assay to further investigate the inhibitory effects of cratoxylumxanthone C on cell migration. As presented in Figure 7, the migration rates of A549 cells were markedly reduced after treatment with cratoxylumxanthone C for 48 h. The migration rates of A549 cells were 57.93% (control), 48.34% (7.5 *μ*M), 42.42% (15 *μ*M) and 17.26% (30 *μ*M), respectively.

Accumulating studies have reported that the migration of tumors is regulated by FAK and downstream proteins. As revealed in Figure 7, the expression of phosphorylated FAK was inhibited by cratoxylumxanthone C, and the expression of FAK was unchanged after treatment with cratoxylumxanthone C. Matrix metallopeptidase 2 (MMP2) is a key protein of FAK signal pathway, and the MMP2 levels are usually considered to be a indicator of tumor metastasis. Western blotting experiments revealed that the treatment of cratoxylumxanthone C caused the decrease of MMP2 expression dose-dependently. All of the protein detection suggested that cratoxylumxanthone C inhibited tumor metastasis *via* regulating crucial proteins of FAK signal pathway.

### Cratoxylumxanthone C possessed *in vivo* anti-tumor effects

**FIGURE 8:**
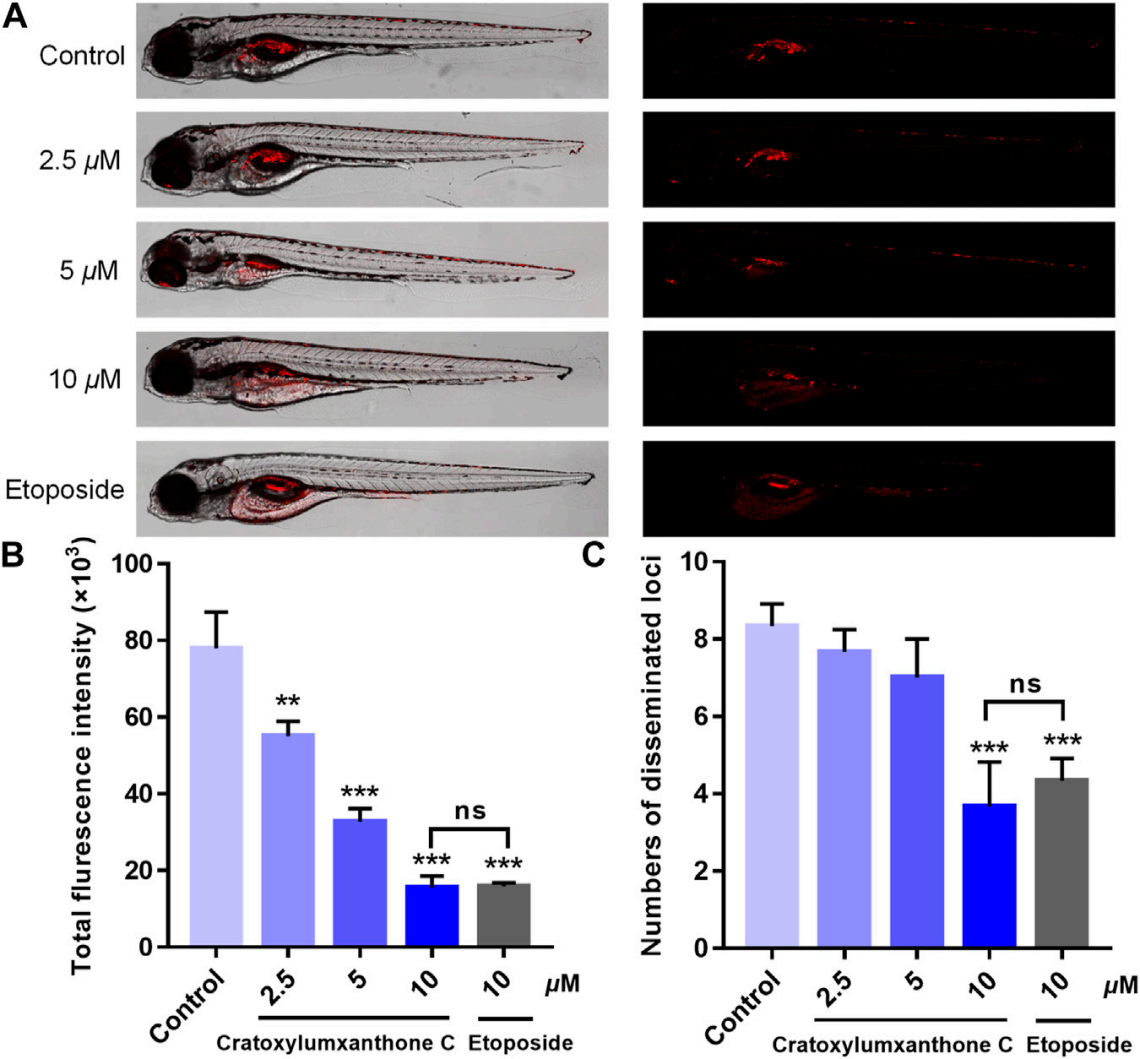
Cratoxylumxanthone C inhibited proliferation and migration of A549 cells in zebrafish xenografts. CM-DiI stained A549 cells were microinjected into 2 dpf zebrafish embryos. After 4 h, tumor-bearing embryos were treated with cratoxylumxanthone C (2.5, 5, and 10 *μ*M) and the positive control, etoposide (10 *μ*M) for 48 h (*n* = 15/group). **(A)** Intensity and distribution of the red fluorescence were imaged under a confocal microscope of disseminated foci in zebrafish. **(B)** The proliferation was quantified by ImageJ software. **(C)** The metastasis of A549 cells were quantified using ImageJ software. All results are expressed as the mean ± SD. **p < 0.01 and ***p < 0.001 versus the control group.

Using zebrafish xenografts, the *in vivo* anti-proliferative and anti-metastatic effects of cratoxylumxanthone C were investigated. The stained cells were microinjected to establish the zebrafish xenograft tumor model and cratoxylumxanthone C was administrated. As revealed in Figure 8A, compared with the blank control, the cell proliferation and migration were significantly inhibited after treatment with cratoxylumxanthone C and the positive control, etoposide. As shown in Figures 8B,C, the relative intensity and foci of red fluorescence decreased with the concentration increase of cratoxylumxanthone C. When the concentration rose to 10 *μ*M, the inhibitory effects of cratoxylumxanthone C were equivalent to those of the etoposide-treated group. Taken together, the zebrafish xenograft experiments indicated that cratoxylumxanthone C had *in vivo* anti-tumor effects by suppressing the tumor proliferation and metastasis.

### Cratoxylumxanthone C blocked angiogenesis of transgenic zebrafish

**FIGURE 9:**
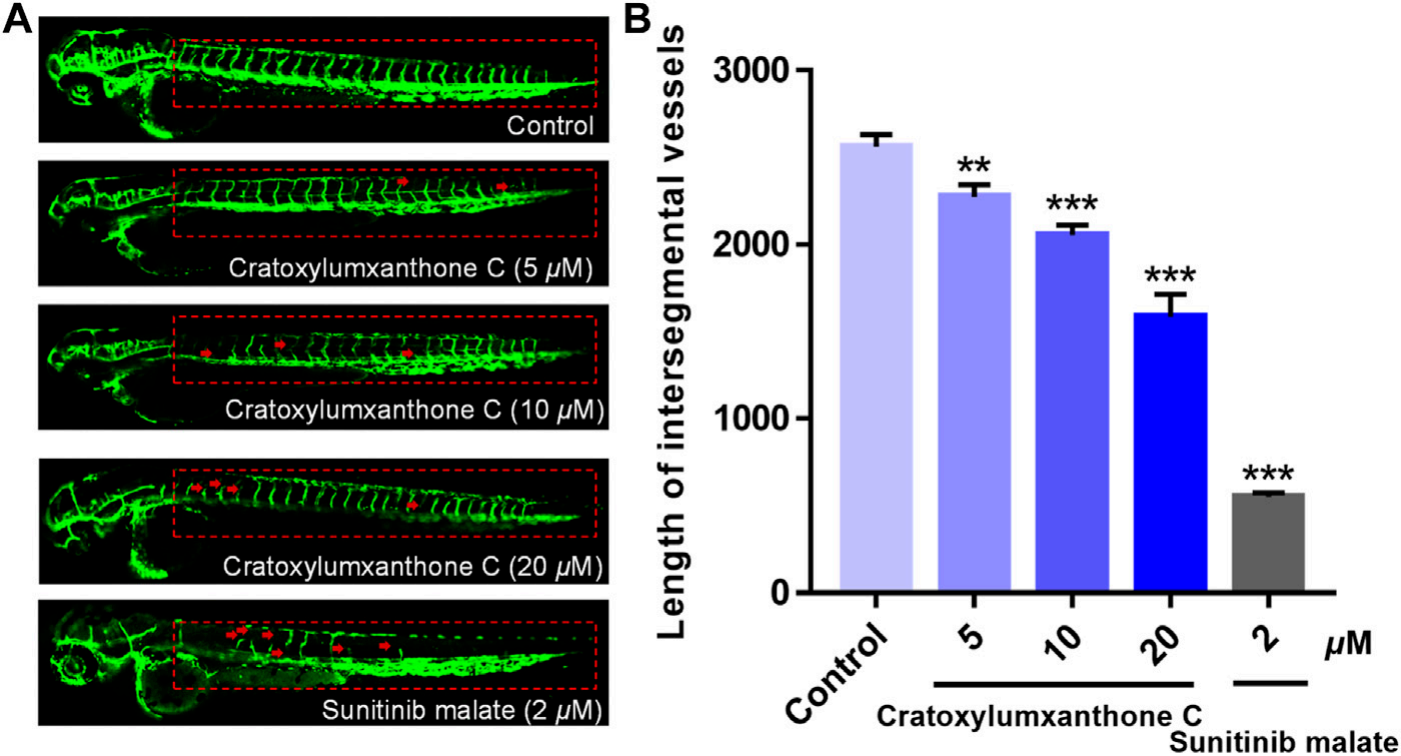
The embryos from transgenic zebrafish *Tg(fli1: EGFP)* were treated with cratoxylumxanthone C (5, 10, and 20 *μ*M) and the positive control, sunitinib malate (2 *μ*M) for 48 h. **(A)** The development of intersegmental vessels (ISVs) was observed under a confocal microscope. **(B)** The average length of ISVs of zebrafish after treating with cratoxylumxanthone C (5, 10, and 20 *μ*M) and sunitinib malate (*n* = 15/group). All results are expressed as the mean ± SD. **p < 0.01 and ***p < 0.001 versus the control group.

In the process of tumor formation and metastasis, angiogenesis is considered as an important boosting factor, providing oxygen and nutrition for tumors. To investigate whether cratoxylumxanthone C affected the angiogenisis, the transgenic zebrafish were used and the newly formed vessels with green fluorescence in zebrafish were detected. As shown in Figure 9A, cratoxylumxanthone C significantly blocked angiogenesis of transgenic zebrafish. As presented in Figure 9B, when the concentration increased from 0 to 20 *μ*M, the length of ISVs from 2,560.19 ± 57.16 *μ*m (control) to 2,274.84 ± 55.81 *μ*m (5 *μ*M), 2053.97 ± 45.91 *μ*m (10 *μ*M), and 1,586.43 ± 103.83 *μ*m (20 *μ*M). The results revealed that cratoxylumxanthone C effectively blocked angiogenesis, which was comparable to the positive control, sunitinib malate (2 *μ*M).

## Discussion

As tumor proliferates abnormally and is highly invasive, the treatment is a great challenge, and new drugs for the treatment of cancer are urgently needed. Natural products including phytochemicals are one of the important sources of new drugs, and more than 40% of new drugs are originated from natural products, such as paclitaxel, camptothecin, and vinblastine, which have been applied in clinical cancer treatment. These successful cases suggest that the discovery of active compounds from natural products is important and crucial in the development of anti-tumor drugs. Natural products with diverse structures and biological activities are an important source of anticancer drug development. It is one of the important strategies of anticancer drug development to discover new anticancer agents from natural products.

Xanthones are a class of heterocyclic compounds with a dibenzo-*γ*-pyrone framework, and it is well known that xanthones and their derivatives have good anticancer activity. According to different substituents, xanthone derivatives can be divided into six categories, simple oxygenated xanthones, glycosylated xanthones, prenylated xanthones, xanthone dimers, xanthonolignoids, and miscellaneous xanthones. Prenylated xanthones are the most studied and widely concerned natural molecules, which exhibit more significant anticancer activity than other xanthones and have broad application prospects as possible and potential anticancer agents (Kurniawan et al., 2021). With the interest in this class of compounds, cratoxylumxanthone C, a prenylated xanthone, was isolated from the plant *C. cochinchinense*, which had the prenyl substituent group, a key functional group for the anticancer activity of xanthone. Cratoxylumxanthone C showed antiproliferative activity in our study, which prompted us to investigate its anti-tumor mechanism and *in vivo* anti-tumor activity.

Tumor proliferation, metastasis, and angiogenesis are key features of tumors, and inhibiting these features of tumors plays an important role in cancer therapy (Li et al., 2021b). In this study, cratoxylumxanthone C was cytotoxic to A549, HepG2, and MCF7 cell lines, having the greatest cytotoxic effects on A549 cells with an IC_50_ value of 17.5 *μ*M. The subsequent mechanistic studies revealed that cratoxylumxanthone C significantly induced apoptosis in A549 cells. It is well known that apoptosis is closely related to ROS and cell cycle (Wang F. et al., 2022). Our further studies showed that cratoxylumxanthone C could increase ROS production and arrest A549 cell cycle in G0/G1 phase. In addition, Western blotting results also showed that the expression levels of apoptosis-related proteins Bcl-2, caspase-3, caspase-9, Mcl-1 and cyclin D1 were decreased.

STAT3 is a transcription factor and intracellular signaling protein that is activated by a variety of growth factors, cytokines, and intracellular kinases (Zou et al., 2020). Previous studies have shown that STAT3 activation is associated with the promotion of tumorigenesis and invasion, and inhibition of STAT3 activation can block oncogene-related pathways (Galoczova et al., 2018). Therefore, STAT3 is recognized as a potential therapeutic target. Our results showed that cratoxylumxanthone C down-regulated the expression of p-STAT3 in A549 cells in a concentration-dependent manner. In addition, the protein levels of Bcl-2, Mcl-1, and cyclin D1 in the STAT3 pathway were decreased dose-dependently. These data suggested that apoptosis of A549 cells was dependent on the inactivation of the STAT3 pathway affected by cratoxylumxanthone C.

In addition to proliferation, migration is also a major feature of tumors. In the current study, the wound healing assay was performed, showing that A549 cells migration was inhibited significantly after treated by cratoxylumxanthone C. FAK, an essential non-receptor intracellular tyrosine kinase, exerts a critical role in migration, and survival (Sun et al., 2016). Furthermore, physiological and pathological tissue remodeling processes, such as wound healing, embryo implantation, tumor invasion, metastasis, and angiogenesis are associated with matrix metalloproteinases (MMPs). Both MMP-2 and MMP-9 are associated with tumor invasion, metastasis and angiogenesis (Stetler-Stevenson, 1999; Kessenbrock et al., 2010; Gonzalez-Avila et al., 2019). According to our study, Western blotting results showed that cratoxylumxanthone C inhibited the expression of p-FAK and MMP-2 in a dose-dependent manner. These data suggested that cratoxylumxanthone C inhibited A549 migration by blocking the FAK pathway.

Cratoxylumxanthone C showed promising anti-tumor activity *in vitro* by modulating STAT3 and FAK pathways. To further confirm its possible application as an anti-tumor drug, we investigated the *in vivo* anti-tumor activity of cratoxylumxanthone C. Recently, Zebrafish tumor-bearing model has become a new tool for tumor biology research and anti-tumor drugs, in which the proliferation and metastasis of tumor can clearly observe (Chen et al., 2021). Cratoxylumxanthone C exhibited dose-dependent inhibitory effects on tumor proliferation and metastasis in zebrafish tumor xenografts. Angiogenesis exerts an crucial function in tumor growth and metastasis, and is closely related to the occurrence, development, invasion and metastasis of malignant tumors (Viallard and Larrivée, 2017; Wang H. et al., 2022). In our study, we demonstrated that cratoxylumxanthone C suppressed angiogenesis in the transgenic zebrafish model.

## Conclusion

The good efficacy and less side effects of phytochemicals have long motivated the search for anti-tumor candidate drugs from natural sources*.* To obtain bioactive molecules as lead compounds to combat cancer, the extract of *C. cochinchinense* was subjected to chromatography for purification and a xanthone, cratoxylumxanthone C, was identified. The cellular study showed that cratoxylumxanthone C inhibited the proliferation and migration of A549 *via* affecting the STAT3 and FAK pathways, in which the mechanistic examination showed that cratoxylumxanthone C suppressed the phosphorylation of STAT3 and regulated the expression of downstream proteins of STAT3, including Bcl-2, Mcl-1, and cyclin D1. At the same time, cratoxylumxanthone C was found to inhibit cell migration *via* the suppression of FAK phosphorylation and down-regulating MMP-2 expression. Angiogenesis, a key sign in tumor formation and development, has been recognized to be closely coupled with tumor proliferation and metastasis. Surprisingly, cratoxylumxanthone C exhibited anti-angiogenesis effects in the transgenic zebrafish model. All of these *in vitro* and *in vivo* experimental results implied that cratoxylumxanthone C, showing excellent anti-tumor activity, can be expected to become a novel chemotherapy drug for NSCLC.

## Data Availability

The original contributions presented in the study are included in the article/Supplementary Material.
