# Supplementary material for: Cratoxylumxanthone C, a natural xanthone, inhibits lung cancer proliferation and metastasis by regulating STAT3 and FAK signal pathways
Source: Front Pharmacol. 2022 Aug 9;13:920422. doi: 10.3389/fphar.2022.920422 (PMC9396379; doi:10.3389/fphar.2022.920422)
Supplement: Supplementary file 1 [file DataSheet1.docx]

**Supplementary Material**

**Cratoxylumxanthone C, a natural xanthone, inhibits lung cancer proliferation and metastasis by regulating STAT3 and FAK signal pathway**

**Materials and methods**

Materials and Cell Culture

Dulbecco’s modified Eagle’s medium (DMEM), Fetal bovine serum (FBS, BI, Israel) were purchased from LABBIOTECH Co. Ltd. (Shandong, China). 3-(4,5-Dimethylthiazol-2-yl)-2,5-diphenyltetrazolium bromide (MTT) and dimethyl sulfoxide (DMSO) were purchased from Solarbio (Beijing, China). Celltracker CM-DiI was obtained from Yeasen Biotechnology Co. Ltd (Shanghai, China). Etoposide was purchased from AdooQ BioScience (Nanjing, China). Sunitinib malate was purchased from Shanghai yuanye Bio-Technology Co. Ltd (Shanghai, China). Annexin V-FITC Apoptosis Detection Kit, ROS Assay Kit, Cell Cycle and Apoptosis Kit, BCA Protein Assay Kit, ECL Detection Kit were provided by Beyotime Biotechnology Co. Ltd (Shanghai, China). Anti-*β*-Actin (Cat# 4970), anti-STAT3 (Cat# 9139), anti-phospho-STAT3 (Tyr 705) (Cat# 9145), anti-caspase-3 (Cat# 9662), anti-caspase-9 (Cat# 9508), anti-Bcl-2 protein (Cat# 14796), anti-cyclin D1 (Cat# 2978), anti-Mcl-1 (Cat# 5453), anti-FAK (Cat# 3285), anti-phospho-FAK (Tyr397) (Cat# 8556) and anti- MMP-2 (Cat# 4022) were all purchased from Cell Signaling Technology (Danvers, MA, USA). Goat anti-rabbit IgG-HRP (Cat# abs20040) and goat anti-mouse IgG-HRP (Cat# abs20001) were purchased from Absin Biotechnology (Shanghai, China).

The cancer cell lines (A549, HepG2, and MCF7 cells) were obtained from Shanghai Institute of Biochemistry and Cell Biology, Chinese Academy of Sciences (Shanghai, China). The cells were cultured in DMEM containing 10% (v/v) fetal bovine serum and 100 U/mL penicillin/streptomycin under a water saturated atmosphere of 95% air and 5% CO_2_.

Zebrafish breeding

Adult AB strain zebrafish were obtained from Shanghai FishBio Co., Ltd. (Shanghai, China). Zebrafish were housed in aerated and UV-sterilized water at 27.5 ± 1 ℃. Zebrafish were fed with artificial fillets and brine shrimp twice daily and housed on a 14 h light/10 h dark cycle. Adult zebrafish were placed in the breeding room overnight and mixed for 30 minutes to obtain embryos. Embryos were cultured in Holt buffer (NaCl 59.9 mM, KCl 0.7 mM, NaHCO_3_ 0.3 mM, and CaCl_2_ 0.9 mM) for further experiments. All processes were performed in accordance with the Animal Ethics Committee of Nankai University.
